# Recent Advances and Challenges in Metal Halide Perovskite Quantum Dot-Embedded Hydrogels for Biomedical Application

**DOI:** 10.3390/molecules30030643

**Published:** 2025-01-31

**Authors:** Junyi Yu, Chengran Zhang, Lijun Kong, Zhengtao Deng

**Affiliations:** 1 College of Chemistry, Jilin University, Changchun 130012, China; yujy1323@mails.jlu.edu.cn; 2 College of Engineering and Applied Sciences, Nanjing University, Nanjing 210023, China; zhangchengran@ntchanged.com (C.Z.); lijun_kong@smail.nju.edu.cn (L.K.)

**Keywords:** metal halide perovskites, quantum dots, hydrogel, biomedical application

## Abstract

Metal halide perovskite quantum dots (MHP QDs), as a kind of fluorescent material, have attracted much attention due to their excellent photoluminescence (PL) quantum yield (QY), narrow full width at half maximum (FWHM), broad absorption, and tunable emission wavelength. However, the instability and biological incompatibility of MHP QDs greatly hinder their application in the field of biomedicine. Hydrogels are three-dimensional polymer networks that are widely used in biomedicine because of their high transparency and excellent biocompatibility. This review not only introduces the latest research progress in improving the mechanical and optical properties of hydrogels/MHP QDs but also combines it with the existing methods for enhancing the stability of MHP QDs in hydrogels, aiming to provide new ideas for researchers in material selection and methods for constructing MHP QD-embedded hydrogels. Finally, their application prospects and future challenges are introduced.

## 1. Introduction

**Figure 1:**
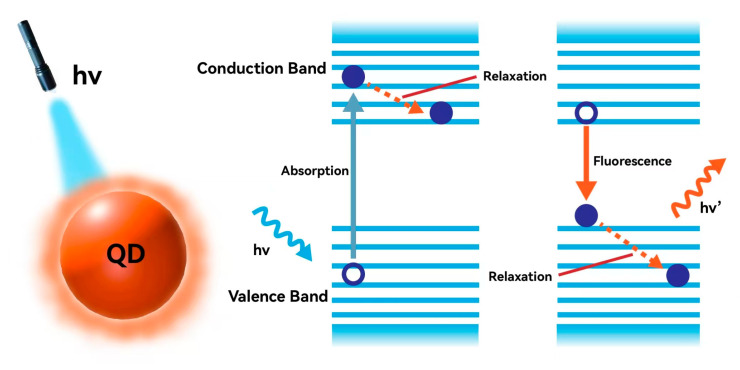
The luminous mechanism of MHP QDs.

Semiconductor nanocrystals, spherical or cubic in shape, with sizes smaller than 20 nm and exhibiting quantum size effects, are referred to as colloidal quantum dots (QDs) [1]. In the electronic energy level structure of QDs, the orbitals filled with electrons form the valence band (VB), while the higher energy empty orbitals constitute the conduction band (CB). Between these two types of bands, the movement of electrons is confined to three dimensions, creating a three-dimensional spherical symmetric potential well. When photons strike the electrons in the energy bands, the electrons are excited and transition to the CB. Simultaneously, holes are generated in the VB, forming electron-hole pairs, also known as excitons. The excited electrons subsequently undergo relaxation, losing some energy, and transition to the lowest energy level in the CB. When the electron transitions from the CB back to the VB under the influence of Coulombic attraction between the electron and the hole, the energy is typically released in the form of photons (Figure 1). As a result, QDs achieve photoluminescence (PL) at specific wavelengths.

The energy of the photons emitted by QDs is determined by the width of the band gap between the VB maximum and the CB minimum, which can be tuned by adjusting the dimensions of the three-dimensional potential well to modify the band gap width. According to the quantum size effect, decreasing the size of the semiconductor crystal widens the band gap, while increasing the size narrows it. Currently, well-established methods exist for calculating the dimensions corresponding to different PL wavelengths [2]. The band gap energy can be approximately described using the Brus Equation (1):

(1) $$E^{*}≃E_{g}+\frac{\hbar^{2}\pi^{2}}{2R^{2}}(\frac{1}{m_{e}}+\frac{1}{m_{h}})-\frac{1.8e^{2}}{\varepsilon R}$$

where, *E_g_* is the band gap of a bulk semiconductor; *R* is the radius of the nanoparticle; and e is the dielectric constant [3,4].

Among these, metal halide perovskite (MHP) QDs have attracted significant attention due to their exceptional optical properties [5]. Their basic chemical composition can be expressed as ABX_3_, where A represents organic/inorganic particles, B denotes metal cations, and X signifies halide anions. Within its internal crystal structure, the metal cation B^2+^ and the halide anion X^-^ form BX_6_^4−^ octahedra. The A^+^ cations occupy the cavities formed between the octahedra. The filling process must satisfy the Goldschmidt tolerance factor t and the octahedral factor μ, which can be calculated as:

(2) $$t=\frac{r_{A}+r_{X}}{\sqrt{2}(r_{B}+r_{X})}$$

(3) $$\mu =\frac{r_{B}}{r_{X}}$$

where r_A_, r_B_, and r_X,_ respectively, refer to the radius of the cations and anions among the chemical constitution of the MHP QDs [6,7]. As for the MHP nanocrystal, when the *t* value is between 0.8 and 1.0, and the *μ* value is between 0.44 and 0.89, the crystal structure is reported to be in a relatively stable state [8].

MHP QDs have demonstrated promising applications in high-definition displays, LED lighting, and solar photovoltaics [9,10]. Despite their excellent optical properties, MHP QDs still face challenges, including biotoxicity and instability in aqueous environments, limiting their applicability in biomedical application.

Hydrogels are generally defined in colloid science as three-dimensional polymer networks containing water molecules and are classified as a type of soft matter. Hydrogel formation mainly occurs in aqueous solutions through physical or chemical interactions that cross-link branched polymer chains, resulting in a three-dimensional network structure. Similar to other gel forms, hydrogels swell in water due to the hydrophilic functional groups present on the polymer chains. These hydrogels absorb water, incorporating water molecules into the three-dimensional network [11]. When the internal pressure from swelling equals the osmotic pressure, swelling equilibrium is achieved, expressed as Equation (4):

(4) $$\prod_{sol}\left(\Phi \right)=G_{0}(\frac{\Phi }{\Phi_{0}}{)}^{\frac{1}{3}}$$

where, the left-hand side represents the osmotic pressure of the solution, while the right-hand side denotes the elastic restoring force of the three-dimensional network resisting swelling [12].

**Figure 2:**
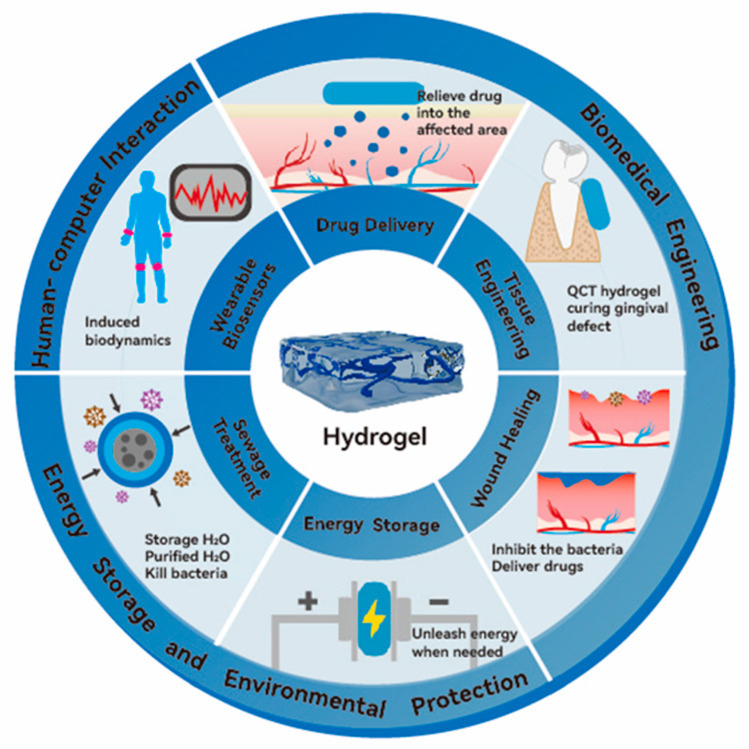
The application prospects of hydrogels.

The three-dimensional network structure and high water content of hydrogels allow them to closely match the internal environment of biological systems, providing excellent biocompatibility [13]. As a result, hydrogels hold considerable promise in biomedical fields, including tissue/soft tissue repair [14,15,16,17,18,19,20], drug delivery [21], and wound dressings [22,23,24,25,26,27,28,29,30,31]. In addition, the outstanding conductivity of hydrogels [32] has broadened their application research in recent years to wearable bioelectronics [33,34,35,36,37], soft robotics, and energy storage materials [38,39,40,41,42,43,44]. Furthermore, hydrogels boast excellent environmental responsiveness [45], expanding their potential in environmental applications such as intelligent waste water treatment [46] (Figure 2).

Since conventional hydrogels inherently lack satisfactory mechanical properties [47], exploring strategies to enhance their mechanical performance has also become a research hotspot in this field. Therefore, comprehensively referencing progress in the research of hydrogels and QDs holds substantial practical significance for improving the performance of hydrogel/MHP QD materials. Florescent hydrogel (HF) hybrid materials have been a widely discussed topic for quite some time, and some articles have systematically reviewed representative work in recent years. For instance, Sayan et al. [48], Koo et al. [49] and Zhang et al. [50] introduced this ‘rising star’ in the field of optical materials in their papers from the perspective of the forming strategies, optical properties, and application prospects that have been reported. Meanwhile, there are limited studies on the performance and feasibility of these composite materials. Hydrogel/MHP QD materials still have significant potential for development to achieve high mechanical properties, biocompatibility, and eventual practical applications.

However, at present, the alternatives to florescent materials are still primarily organic fluorochromes, and nano particles like graphene QDs(GQDs) [51], carbon QDs(CDs) [52], or semiconductor QDs like CdTe [53]. It is noteworthy that only a handful of attempts to integrate MHP QDs into hydrogels have been made so far. Due to the good biocompatibility and transparency of hydrogels, hydrogels and MHP QDs seem to have complementary properties, suggesting the potential application of their composite materials in in vivo and in vitro medical imaging. This concept of integrating MHP QDs into hydrogel matrices has attracted researchers’ interest in recent years [51,52,53]. To date, the development of hydrogel/MHP QD materials is still in its infancy. Research on the preparation of hydrogel/MHP QD materials has mainly focused on improving the stability of MHP QDs in aqueous and air media [54,55,56,57].

In this review, we first summarize the current research status of hydrogel/MHP QD flexible materials, and then introduce recent advances in improving the mechanical properties of hydrogels, highlighting representative preparation strategies. Next, we discuss stability and toxicity issues of MHP QDs in aqueous and air media and the progress in research on methods to address these problems. Finally, we summarize design considerations for enhancing the performance and expanding the practical applications of hydrogel/MHP QD materials, aiming to provide researchers with more systematic perspectives and considerations for future studies in this field.

## 2. Research Advances of Hydrogel/MHP QD Materials

The concept of utilizing hydrogels as a working platform for MHP QDs has gained attention from researchers in the past few years [51,52,53,54,55,56,57]. Current studies mainly focus on developing flexible sensing materials. Most reported preparation methods involve mixing a processed and relatively stable MHP QD solution with a polymer or its precursor solution used for hydrogel synthesis, followed by cross-linking to form hydrogel/MHP QD materials. In general, the current methods for characterizing hydrogels/MHP quantum dots are as follows: (1) optical properties, such as photoluminescent quantum yield (PLQY), photoluminescent lifetime, and absorption spectrum; (2) structural and morphological properties, such as X-ray diffraction (XRD), transmission electron microscopy (TEM), and dynamic light scattering (DLS); (3) mechanical properties, such as Young’s modulus, tensile strength, and elongation at break, which characterizes the stiffness or rigidity of the material.

**Figure 3:**
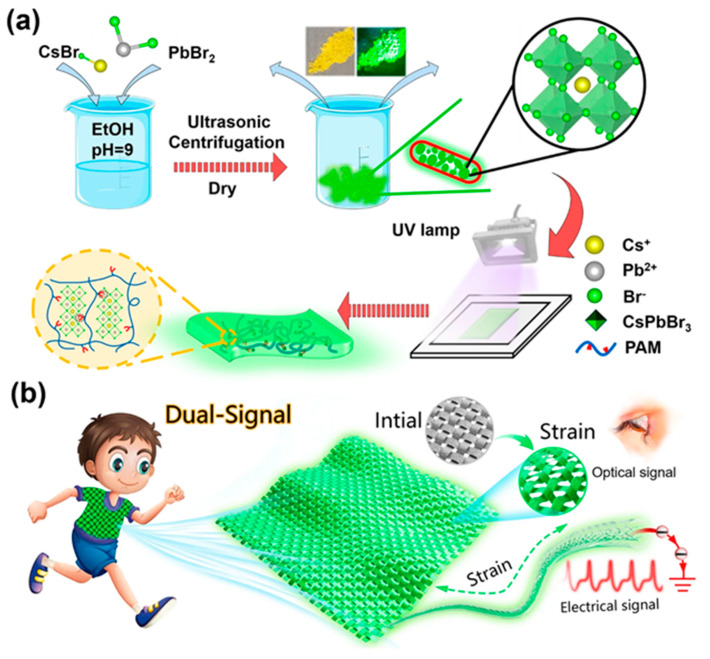
The application of visual-digital strain sensors with stretchable luminescent perovskite-polymer hydrogels. (**a**) Schematic diagram of the synthesis process of perovskite-based fluorescent hydrogels. (**b**) The design concept of the stretchable visual-digital strain sensor textile. Reprinted with permission from ref. [54]. Copyright 2023 Springer Nature.

Li et al. [54] explored embedding CsPbBr_3_@PbBr(OH) (CPB@PBOH), which features water stability due to the formation of a PbBr(OH) protective shell after ethanol immersion, into a polyacrylamide (PAM) hydrogel matrix. The resulting CPB@PBOH-PAM hydrogel demonstrated the ability to withstand a maximum strain of approximately 740%, reaching 51.3 kPa, while maintaining a narrow-band emission spectrum. The fluorescence intensity did not significantly decrease compared to CPB@PBOH without the hydrogel matrix. In further studies, they added a black carbon nanotube (CNT) thin film as a masking layer on the material surface, enabling fluorescence intensity to vary with strain. This demonstrated the potential of such materials to be processed into flexible textiles for applications in motion detection and underwater interaction scenarios (Figure 3).

Liu et al. [55], in their study, encapsulated CsPbBr_3_ QDs within polydimethylsiloxane (PDMS) microspheres via an emulsion method and demonstrated their improved water stability and acid-base tolerance compared to their parent QDs. Subsequently, the authors attempted to integrate CsPbBr_3_@PDMS microspheres into polyacrylamide (PAAm) hydrogels, successfully synthesizing hydrogel/CsPbBr_3_@PDMS composite materials. The experimental results reported that the composite could withstand a maximum strain of approximately 1000%, and this method had a minimal impact on the photoluminescence quantum yield of CsPbBr_3_ MHP QDs. Furthermore, the study highlighted the potential of these materials for applications in flexible optoelectronic devices. Similarly, Zhang et al. [56] reported that CsPbBr_3_@PI microspheres could also enhance the stability of MHP QDs; however, no studies have yet investigated their optical properties when integrated into a hydrogel matrix.

**Figure 4:**
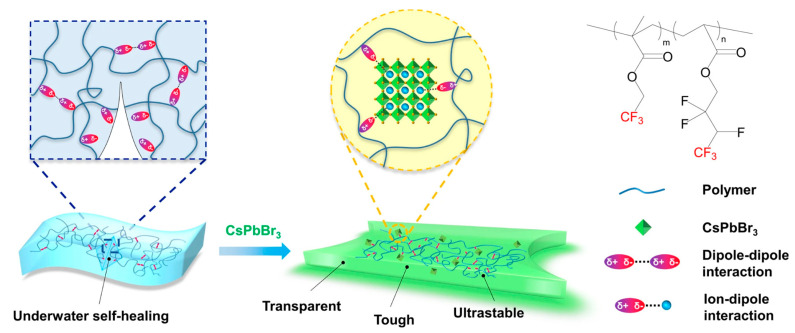
The design of the all-dipole fluorine elastomer using the copolymer of 2,2,2-trifluoroethyl methacrylate (TFEMA) and 2,2,3,4,4,4-hexafluorobutyl acrylate (HFBA) to protect the QDs (namely, TFE-HF-QD). Reprinted with permission from ref. [57]. Copyright 2022 Springer Nature.

Notably, Liu et al. [57] did not pre-encapsulate the CsPbBr_3_ QDs; instead, they selected a polymer with trifluoromethyl (TFE) groups at the end of its side chains as the material for synthesizing the hydrogel matrix (Figure 4). The authors demonstrated that the dipole–dipole interactions between the trifluoromethyl groups on the side chains and the NCs significantly improved the water stability and acid-base tolerance of the encapsulated NCs. The composite material synthesized in this study exhibited an average fluorescence lifetime of 20.59 ns, with a maximum strain of up to 1300% and a fracture energy of 30 kJ·m^−2^. This represents one of the few hydrogel/MHP QD materials to date that combines high mechanical performance with excellent optical properties.

**Figure 5:**
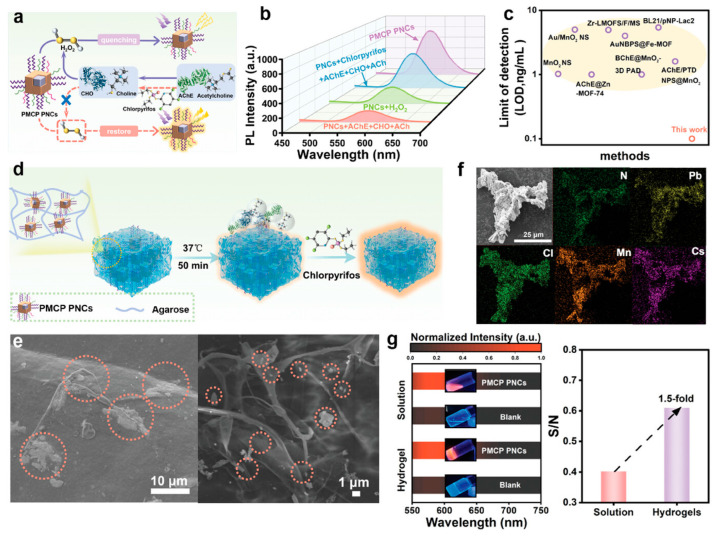
(**a**) Schematic diagram of sensing for monitoring of organophosphorus pesticides. (**b**) Feasibility of sensing principle. (**c**) Comparison of detection limit of sensing platform with other methods. (**d**) Schematic diagram of PMCP PNC-based hydrogel-sensing platform. (**e**) SEM image of PMCP PNC-based hydrogel surface (**left**) and intersecting surface (**right**). The circles in subfigure are for PNCs. (**f**) Element mapping of PMCP PNC-based hydrogel. (**g**) Comparison of PL intensity between liquid-phase sensing platform and hydrogel-sensing platform. Reprinted with permission from ref. [58]. Copyright 2024 WILEY.

Recently, Li et al. [58] studied NH_2_-PEG-COOH-capped Mn^2+^: CsPbCl_3_/CsPb_2_Cl_5_ (PMCP) core/shell hetero perovskite QDs, processed with trinity strategies for enhanced stability and water solubility, into an agarose hydrogel matrix using a “one-pot” method. Based on the inhibitory effect of Dursban (a type of pesticide) on the cascade enzyme of acetylcholinesterase (AChE) and choline oxidase (CHO), as well as the fluorescence intensity changes of PMCP PNCs in response to different H_2_O_2_ concentrations, they designed a hydrogel/PMCP PNCs material as a high-sensitivity flexible sensor to monitor pesticide residues in food (Figure 5).

Previously, research on hydrogel/QD fluorescent gels mainly focused on the doping of carbon QDs [59,60,61,62] or graphene QDs [63]. Progress in hydrogel/MHP QD materials remains relatively limited. Current studies primarily aim to develop more stable and water-soluble MHP QDs, and treat hydrogels mainly as a substrate for MHP QDs to facilitate practical applications. Thus far, hydrogel/MHP QD materials have not achieved significant advancements in mechanical properties, which limits the development of their application scenarios [64].

## 3. Challenges and Strategies for Improving Mechanical Properties of Hydrogels

As a polymeric material, hydrogels are prone to structural damage mainly due to the stress concentration in certain areas, leading to cracks. According to the Lake–Thomas theory, crack propagation under continuous stress first destroys the polymer chains in the crack’s forward region, which then transmits the damage; eventually, the notch turns into a running crack [65], causing the material to fracture.

The mechanical property of hydrogels, a major factor determining their practicality, mainly depends on their fracture resistance, rigidity, hysteresis effect, and sensitivity to notches. Fracture resistance is evaluated through the maximum stress max, maximum strain max, and fracture energy. Rigidity is primarily determined by the hydrogel’s Young’s modulus, which can be approximately calculated as Equation (5):

(5) $$E=\frac{\Gamma }{\lambda }$$

This value is typically derived from the slope of the stress–strain curve fitted from experimental data. Generally, a higher Young’s modulus indicates greater rigidity, while a lower Young’s modulus implies weaker rigidity and better ductility. Hydrogels with different Young’s modulus values can be tailored for various applications.

The hysteresis effect refers to the ability of a hydrogel to recover its original shape during unloading after being subjected to stress. Experimentally, the hysteresis effect of hydrogels is visually represented by the area enclosed by the loading–unloading curve. Specifically, the smaller the area, the weaker the hysteresis effect, indicating stronger shape recovery capability. Research in this field has long focused on developing high-performance hydrogels with high fracture energy, low hysteresis, and simple preparation processes [66,67]. Current progress is mainly made in designing hydrogels with specific three-dimensional internal structures and identifying polymers with better mechanical properties.

### 3.1. Double-Network Hydrogels

Double-network hydrogels (DN hydrogels) typically consist of a densely cross-linked, mechanically rigid first network and a loosely cross-linked, soft, and highly stretchable second network. These two networks interact synergistically: the rigid/brittle first network acts as a sacrificial unit that effectively dissipates energy under loading, while the low cross-link density and high toughness of the second network allow it to withstand large strains [68,69] and transfer the stress to the first network. This synergistic interaction between the two networks is the major reason why DN hydrogels can have improved mechanical property [70].

The concept of DN hydrogels was first reported by Gong et al. in 2003 [71]. They used poly(2-acrylamido-2-methylpropanesulfonic acid) (PAMPS) and poly(acrylamide) (PAAm) as the raw materials for the first and second networks, respectively, to prepare a DN hydrogel. This DN hydrogel demonstrated significant advancements in mechanical properties compared to the then-contemporary interpenetrated network (IPN) hydrogels [72] and fiber-reinforced hydrogels [73]. Subsequently, researchers in the same group, such as Yasuda et al., developed DN hydrogels with excellent wear resistance, including PAMPS-PDMAAm gels composed of poly(2-acrylamido-2-methylpropanesulfonic acid) and poly(N,N’-dimethyl acrylamide), and cellulose/PDMAAm gels consisting of bacterial cellulose and poly(dimethyl acrylamide) [74].

However, studies by Na et al. [75] and Kawauchi et al. [76] revealed that DN hydrogels have a pronounced “necking phenomenon” under stress. Yu et al., using images captured with an optical microscope and a color three-dimensional violet laser scanning microscope, observed local damage zones on the surface of DN hydrogels under stress [77]. Gong et al. explained this as being due to the high cross-link density of the first network, where covalent bonds break irreversibly under stress to dissipate energy [78,79]. This characteristic leads to an obvious hysteresis effect in DN hydrogels after loading. To address this issue, Suo et al. synthesized ionically and covalently cross-linked DN hydrogels, using calcium ions as the cross-linking agent for the ionically cross-linked alginate first network and N,N′-methylenebisacrylamide as the cross-linker for the covalently cross-linked polyacrylamide second network. Since the ionic bonds in these DN hydrogels served as sacrificial units that reformed more rapidly than covalent bonds, these hydrogels had a very low hysteresis effect during loading–unloading experiments [65]. Based on similar principles, Chen et al. synthesized fatigue-resistant DN hydrogels using the two-step method, such as Agar/PAMAAc-Fe^3+^ DN gels [80]. Li et al. employed a dual ionic cross-linking strategy to synthesize Agar/PAMAAc-Fe^3+^ DN gels, reporting high strength and toughness [81]. These approaches demonstrate that the incorporation of reversible non-covalent bonds as sacrificial units effectively enhances the mechanical properties of DN hydrogels.

**Figure 6:**
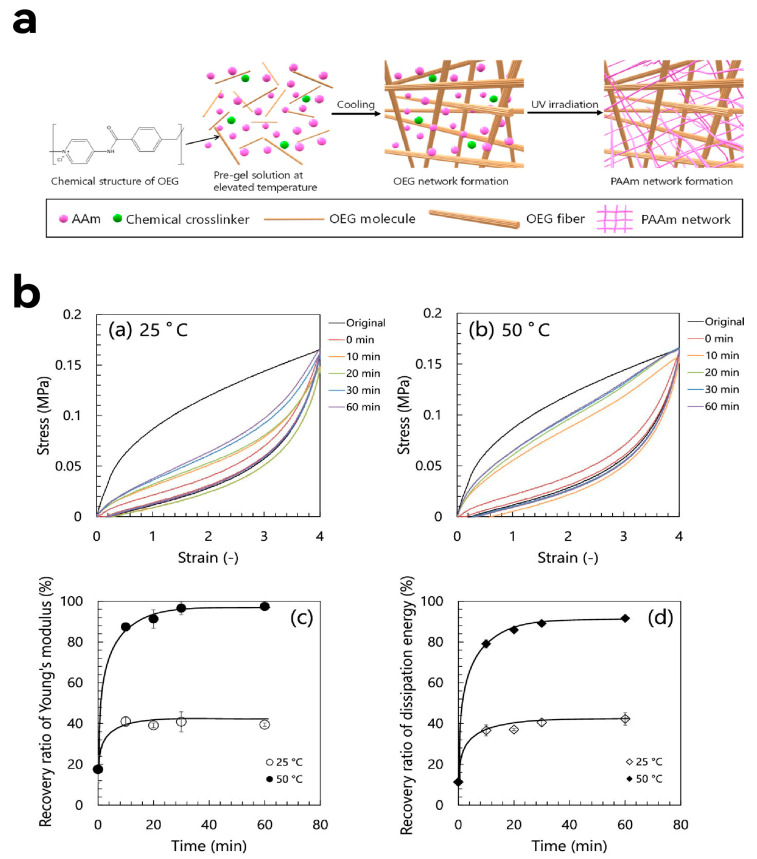
(**a**) Schematic of One-Pot Sequential Synthesis of OEG/PAAm Double-Network Hydrogels. (**b**) Self-healing properties of the sacrificial network in the OEG/PAAm DN hydrogel. (**a**,**b**) Second cyclic loading–unloading curves of specimens annealed at 25 °C (**a**) and 50 °C (**b**) for different times after first cycle. The curves marked “original” are first cyclic loading–unloading curves. (**c**,**d**) Waiting time dependence of recovery ratios of Young’s modulus (**c**) and dissipation energy (**d**) of OEG/PAAm DN hydrogels. The OEG content and MBAA concentration in gels are 5 wt.% and 0.1% of AAm in moles, respectively. Measurements are performed at 25 °C. Reprinted with permission from ref. [96]. Copyright 2022 American Chemical Society.

In recent years, researchers have enriched the range of reversible non-covalent bonds. Components such as helical physical cross-links [82], crystallites serving as physical cross-links [83], nanoclusters [84], metal–ligand coordination bonds [85], hydrogen bonds [86,87,88], silicon-particle networks [89,90,91], and reversible covalent peptide bonds regulated by mechanocoupled enzymatic reactions [92] have been validated as effective choices for reversible non-covalent components. Although the introduction of reversible sacrificial units mitigated the issue of permanent deformation in DN hydrogels after loading, experimental data from multiple studies reveal that DN hydrogels still have significant strain rate dependency. Faster strain rates lead to more obvious hysteresis effects [93,94,95]. To address this, Yasui et al. proposed optimizing DN hydrogels by using thixotropic gels as the first cross-linked network [96]. They utilized quasi-solid-state thixotropic hydrogels composed of an oligomeric electrolyte gelator (OEG), which has unique rheological behavior (Figure 6). This represents a significant breakthrough in recent research on improving the mechanical properties of DN hydrogels. Currently, the design of DN hydrogels remains a widely discussed topic in the development of high-performance hydrogels.

### 3.2. Sliding-Ring Hydrogels (SR Hydrogels)

Beyond enhancing the mechanical properties of hydrogels through synergistic interactions between the two types of cross-linked networks, much attention has also been paid to designing and modifying the three-dimensional topological structure of polymer chains in hydrogels. Sliding-ring hydrogels utilize the “pulley effect” generated by their internal topological structure, spreading stress across the entire network.

In 2001, Okumura et al. [97] synthesized a hydrogel termed the “polyrotaxane gel”, which achieved over 95% transparency, withstood stress in the range of 1500–2000 Pa, endured 200% strain, and demonstrated remarkable stability for long-term storage. This hydrogel was synthesized using polyrotaxane—a representative polymer with sliding rings, comprising a high-molecular weight polyethylene glycol (PEG) chain encircled by α-cyclodextrin (α-CD) and capped with bulky end groups—as the polymer and chloro-s-triazine as the cross-linker [98,99].

Through comparative experiments with hydrogels synthesized from mixtures of PEG, α-CD, and chloro-s-triazine, they confirmed that the improved mechanical properties of the hydrogel were attributable to its unique structural feature: cyclic molecules encircling the polymer chains. During gelation, PEG chains were cross-linked by α-CDs along their length, forming a figure-eight-like topology akin to a pulley system, linking the long-chain polymers. This unique topology was the key to the “pulley effect”, which distributed stress across the polymer chains, significantly enhancing the hydrogel’s fracture energy. Moreover, the sliding mobility of α-CDs along the PEG chain considerably improved the hydrogel’s extensibility [97].

**Figure 7:**
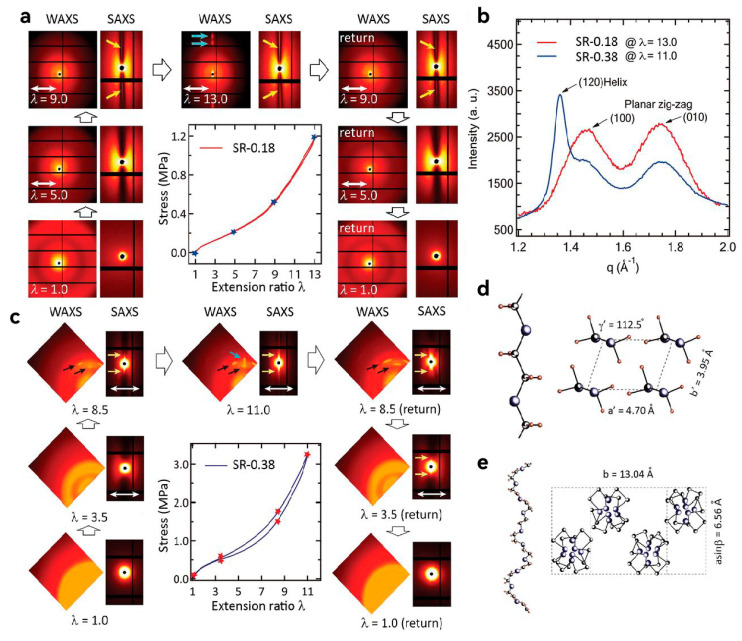
(**a**,**b**) WAXS and SAXS patterns of SR0.18 (**a**) and SR-0.38 (**b**) during a loading–unloading cycle. The white double arrows denote the stretching direction. (**c**) WAX profiles of SR-0.18 and SR-0.38 gels in the direction perpendicular to stretching. (**d**) Structure of planar zigzag PEG and its triclinic crystal. (**e**) Structure of 7/2 helix PEG and its monoclinic crystal. Reprinted with permission from ref. [102]. Copyright 2021 AAAS.

In addition, the stretching process often results in chain breakage, causing hysteresis effects [100]. The synthesis of polyrotaxane-based hydrogels reported in this study provides an innovative approach for designing the spatial structure of single-network hydrogels. In 2021, Ito, a collaborator in the above-mentioned research, and his colleagues further optimized this approach. Liu et al. similarly employed polyrotaxane as the polymer containing sliding rings and formed the topological structure through cross-linking between the rings (Figure 7). However, they hypothesized that the number of sliding rings on the PEG chain would influence the toughness of these SR hydrogels. Fewer rings allowed for greater sliding mobility, which can effectively increase the extension and interaction of the PEG chains [101]. Consequently, Liu et al. intentionally reduced the α-CD content during synthesis, achieving a coverage ratio of only 2% on the PEG chains. The resulting SR hydrogels could withstand stresses of 6.6–22 MPa and display near-complete recovery after stretching. Moreover, these SR hydrogels demonstrated exceptional resistance to crack propagation. Compared with tetra-PEG hydrogels and the first-generation SR hydrogels, these optimized SR hydrogels showed visibly superior mechanical properties [102]. The authors explained this obvious improvement, which results solely from adjusting the number of rings, using small-angle neutron scattering (SAXS). The reduced number of rings increased the exposed portions of the PEG chains. During stretching, aligned PEG chains were closely packed together, leading to a reversible “strain-induced crystallization” phenomenon, which significantly impeded crack propagation [103,104]. Although this study did not introduce a methodological breakthrough, it revealed a previously unconsidered interaction between polymer chains, providing a new dimension for designing SR hydrogels with high mechanical performance.

Since the advent of the first-generation SR hydrogels, many researchers have drawn inspiration from this topological structure, expanding the available synthesis methods and application fields of SR hydrogels based on the fundamental structure of polyrotaxanes (“strings” encircled with “rings”). For instance, Tang et al. [105] introduced the concept of “pseudo-slide-rings”, and constructed dynamic topological structures through hydrogen-bond-mediated cross-linking of α-CDs.

The emergence of computational chemistry is a potential tool for guiding SR hydrogel design. For example, Zhang et al. investigated the factors influencing the mechanical properties of SR hydrogels using molecular dynamics (MD) simulations. In their study, they constructed coarse-grained bead-spring models to approximate the SR hydrogel structure within the large-scale atomic/molecular massively parallel simulator (LAMMPS) [106], and simulated various mechanical behaviors, including stretching-recovery, compression-recovery, and viscoelasticity. This research provided molecular insights into the slide-ring junction structure of SR hydrogels [107].

Currently, studies utilizing computational chemistry and material simulation software to assist in the topological design of hydrogels remain limited. However, we believe that theoretical calculations can serve as a valuable tool for guiding the topological design of hydrogel materials in the future, representing a promising direction. Thanks to their unique spatial topology, SR hydrogels dissipate stress without relying on sacrificial units. Consequently, SR hydrogels can rapidly recover their original shape after unloading without polymer chain damage, making them excellent candidates for medical materials. To date, optimizing the structural design and preparation methods of SR hydrogels remains a hot research topic. Identifying more suitable long-chain polymers to further simplify the internal three-dimensional structure and rationally designing functional materials with SR hydrogels are the challenges currently faced in this field.

### 3.3. Topological Entanglement of Polymer Chains

It has long been recognized that polymer chains in elastomers can form entanglements [108]. However, the effects of entanglement on the fracture energy, fatigue, and other mechanical properties of hydrogels have only recently captured the attention of researchers. This approach is part of the broader strategy of topo-architecting polymer networks to enhance their mechanical properties.

Kim et al. [109] discovered that when the density of entanglements among polymer chains significantly exceeds the cross-linking density, single-network hydrogels can achieve high toughness without being apparently embrittled. In this study, they synthesized highly entangled hydrogels by cross-linking long-chain, high-molecular-weight polyacrylamide (PAAm) with an extremely low ratio of water, cross-linker, and initiator. Comparative tests revealed that these hydrogels could withstand stresses of nearly 400 kPa, which is far higher than those of regular hydrogels with the same components. In addition, these hydrogels had little hysteresis effects and were virtually unaffected by strain rates. Furthermore, the significantly lower friction coefficient of these hydrogels compared to other regular elastomers demonstrated that the entangled polymer chains could still slip freely. This research proposed an alternative approach to improving the mechanical properties of hydrogels without relying on sacrificial units to dissipate stress, as seen in SR hydrogels. The innovation lies in substituting dense entanglements for a majority of cross-links, such that stress can be evenly distributed throughout the network. Under such conditions, the method and intensity of cross-linking have a greatly reduced impact on the hydrogel’s mechanical properties. In other words, as long as the internal long polymer chains do not break, the internal structure can recover upon unloading. This concept provides a novel solution to the “stiffness–toughness conflict”.

Shortly thereafter, Nian et al. [110] refined the preparation methods for highly entangled hydrogels based on this principle. In their study, they proposed a technique referred to as the “kneading dough” method. Specifically, they used a photoinitiation technique for cross-linking [111], where high-molecular-weight PEG (MV = 8 × 10^6^ g mol^−1^) and a low ratio of benzophenone photo initiator (benzophenone ratio B = 3.2 × 10^−4^) were thoroughly mixed in isopropanol (IPA) solvent. After a process of drying, compacting, and baking to remove the solvent, a “dough-like” elastomer was obtained. By kneading the material to achieve sufficient entanglement among the PEG chains, followed by photochemical cross-linking and swelling, they formed a hydrogel. The resulting hydrogel could withstand stresses approaching 8 MPa and demonstrated excellent fatigue resistance. At present, research on enhancing the mechanical properties of hydrogels using this method remains limited, indicating promising opportunities for further investigation.

### 3.4. Phase-Separation-Induced Formation of Heterostructure (PS Hydrogels)

The phase separation of polymers refers to the thermodynamically driven process in which polymers self-assemble to reduce system energy within a mixed hydrogel system composed of two polymers with differing compatibility. The energy of the mixed system can typically be calculated using the Flory–Huggins function (6):

(6) $$\Delta {\bar{F}}_{mix}=\Delta {\bar{U}}_{\mathrm{mix}}-T\Delta {\bar{S}}_{mix}=kT\left[\frac{\phi }{N_{A}}\ln\phi +\frac{1-\phi }{N_{B}}\ln\left(1-\phi \right)+\chi \phi \left(1-\phi \right)\right]$$

where, ϕ is the volume fraction of each component; NA and NB are the lattice points occupied by the two polymers in the system; and *χ* is the Flory–Huggins interaction parameter used to describe the interaction between the two phases.

When the concentrations of the two polymers in the system are sufficiently high, simple mixing leads to instability due to compatibility differences. Whether phase separation occurs can be roughly determined by the sign of the second derivative of the Flory–Huggins function with respect to the polymer volume fraction (7):

(7) $$\frac{\partial^{2}\Delta {\bar{F}}_{mix}}{\partial \phi^{2}}=kT\left[\frac{1}{N_{A}\phi }+\frac{1}{N_{B}\left(1-\phi \right)}\right]-2\chi kT$$

This information can be visually interpreted through the convexity of the free energy-volume fraction ( - ) curve [112].

It has long been observed that interactions among densely packed polymers are linked to improved mechanical properties in elastomers [113,114]. Based on this principle and the water-containing characteristics of hydrogels, researchers have explored phase separation methods. Sato et al. [115] first demonstrated that heterostructured hydrogels formed via phase separation have significantly enhanced mechanical properties compared to conventional hydrogels. In their study, PAAm hydrogels were immersed in a DMF/water solution—where DMF is a poor solvent for PAAm and water is a good solvent. A series of orthogonal experiments were conducted by varying the concentration of DMF (CDMF). The sharp decrease in hydrogel transparency observed when CDMF reached 80% confirmed the occurrence of phase separation. Sato et al. observed that when CDMF exceeded a certain range, the hydrogels became brittle. This was attributed to excessive phase separation, where strong polymer–polymer interactions within the solvent-poor phase (glassy state) prevented these regions from acting as sacrificial units to dissipate stress, resulting in catastrophic failure [115]. This phenomenon highlights a major challenge for phase-separated hydrogels: the lack of interactions between phases makes phase separation difficult to control, and the material faces significant hysteresis over time. To address this, Zhang et al. [116] immersed a rubbery elastomer formed from hydrophobic poly(ethyl acrylate) (PEA) into a precursor solution containing amphiphilic acrylic acid monomers (AAc), the photoinitiator Irgacure2959, and the cross-linker N,N′-Methylenebisacrylamide (MBAA). The precursor solution infiltrated the PEA cross-linked network, and subsequent photoinitiated free-radical polymerization and cross-linking triggered phase separation. This hydrogel could withstand stress levels approaching 7 MPa with negligible residual strain upon unloading.

Moreover, researchers have discovered that phase-separated hydrogels have superior viscoelastic properties [117]. Cui et al. [118] compared the adhesion strength of phase-separated hydrogels prepared by immersion in ethanol/water solutions (poor/good solvent mixtures) with that of uniformly swollen PAAm hydrogels immersed in water. The phase-separated hydrogels had significantly enhanced viscoelasticity. This improvement was attributed to the substantial increase in polymer density within both the interior and surface of the hydrogels due to phase separation, which increased the number of polymer chains per unit area in contact with solid surfaces, enhancing physical interactions at the gel–solid interface. Combined with the toughening effect of sacrificial units within the phase-separated gels, this synergy contributed to the remarkable viscoelasticity of phase-separated hydrogels.

## 4. Challenges and Strategies for Enhancing the Stability of MHP QDs

It is well known that, due to their crystal structure, MHPs have relatively poor stability, and are prone to degradation, which causes defects on the crystal surface. These defects, in turn, adversely affect the long-term stable photoluminescence of MHPs. Currently, one of the major challenges faced by MHPs is their instability in air and humid environments [119,120]. This limitation hinders the full utilization of their advantages, such as high PLQY and tunable fluorescence wavelengths, in many application scenarios.

Due to the high-water content of hydrogel materials and their inability to effectively block oxygen infiltration, it is necessary to identify suitable pretreatment methods to improve the stability of QDs. These considerations are important in the development of high-mechanical-property hydrogel/MHP QD composite materials. Enhancing the stability of MHPs can significantly extend their fluorescence lifetime. Nie et al. [121] conducted a systematic classification and review in 2022 of methods proven to improve the environmental stability of MHP QDs. Moreover, Sanjayan et al. [122] reviewed the specific mechanisms by which various environmental factors impact MHP QDs and corresponding solutions, providing guidance for recent research directions. Currently, the primary approach to enhancing the water solubility and anti-corrosion properties of MHP QDs involves constructing core/shell structures to encapsulate the QDs. This section summarizes recent progress in this area.

### 4.1. Semiconductor Shell Encapsulation

**Figure 8:**
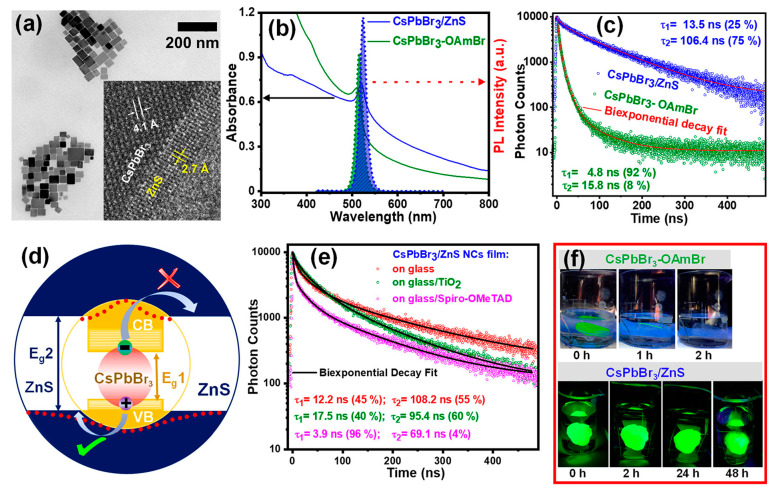
(**a**) TEM image of CsPbBr_3_/ZnS core/shell NCs showing cubic morphology. Inset shows HRTEM image of an NC near the core/shell interface. (**b**) UV–visible absorption and PL spectra showing little red shift for CsPbBr_3_/ZnS core/shell NCs. (**c**) Comparison of PL decay shows a huge increase in PL lifetime for CsPbBr_3_/ZnS core/shell NCs. (**d**) Schematic showing pseudo type-II band alignment at the CsPbBr_3_/ZnS core/shell interface, where the electron is confined inside the core but the hole is delocalized over both core and shell. (**e**) PL decay of CsPbBr_3_/ZnS core/shell NCs film deposited on glass, TiO_2_, and spiro-OMeTAD. (**f**) Digital photographs of the films of CsPbBr_3_/ZnS core/shell NCs and CsPbBr_3_–OAmBr NCs dipped in beakers full of water and excited with UV lamp (365 nm). Reprinted with permission from ref. [123]. Copyright 2020 American Chemical Society.

For core-shell (CS) structure MHP QDs, the thermal injection synthesis process that is successful in II-VI and III-V semiconductor QD systems encounters difficulties. This is mainly due to lattice incompatibility and the stringent conditions required for shell formation. The soft ionic lattice of perovskite leads to increased ion mobility, which raises the risk of ion exchange between the “core” and “shell”, complicating the realization of the CS structure. The basic ionic crystal nature of perovskite results in common surface defects. These defects can cause non-radiative quenching, reducing the overall efficiency. Although the growth of thin oxide inorganic materials like SiO_2_ or Al_2_O_3_ was designed to protect MHP QDs from oxidation, it is often accompanied by unexpected oxidation, leading to irreversible degradation. To solve this problem, Ravi et al. [123] utilized zinc diethyldithiocarbamate (Zn(DDTC)_2_) as a precursor for ZnS and synthesized a uniformly coated CsPbBr_3_@Zn(DDTC)_2_ core/shell structure by treating CsPbBr_3_ with Zn(DDTC)_2_ at 120 °C. Experimental results verified that the fluorescence quantum yield (PLQY) of the structure remained stable in both aqueous media and air (Figure 8).

Furthermore, materials such as PdS [124], CdS [125], and PbSO_4_-oleate [126] have also been explored as semiconductor shell components. However, these studies mainly demonstrated the extension of fluorescence lifetimes in air, with limited discussion on their water solubility and stability in aqueous environments.

### 4.2. Oxide or Hydroxide Shell Encapsulation

The use of mesoporous silica as a coating layer for MHP QDs was first reported by Wang et al. [127]. This method builds upon earlier techniques of silica surface coating on ODs [128], offering a more efficient and simplified solution. In recent years, further advancements in this approach have been achieved.

Carulli et al. [129] attempted to directly grow CsPbX_3_ in situ within the pores of mesoporous silica nanospheres (SiO_2_-NSs) by employing a solid-state constrained growth method, synthesizing various CsPbX_3_-SiO_2_ core/shell materials. They demonstrated the stability of these materials in aqueous and strongly acidic solutions by analyzing changes in radioluminescence (RL) and photoluminescence (PL) intensities. Interestingly, their study revealed that radiative emissions from CsPbX_3_, triggered by X-ray excitation, could convert triplet oxygen in aqueous solutions to singlet oxygen—a cytotoxic radical capable of destroying tumor cells. This finding highlights the potential of these materials for precision radiotherapy in oncology, reducing damage to healthy cells. To further enhance the stability of MHP/SiO_2_ composites, Zhong et al. [130] recently synthesized CsPbBr_3_@MSNs-PbBr(OH)/SiO_2_ materials with a dual-layer protective shell composed of PbBr(OH) and mesoporous silica. They also investigated its potential application in miRNA detection.

### 4.3. Multilayer Shell Encapsulation

**Figure 9:**
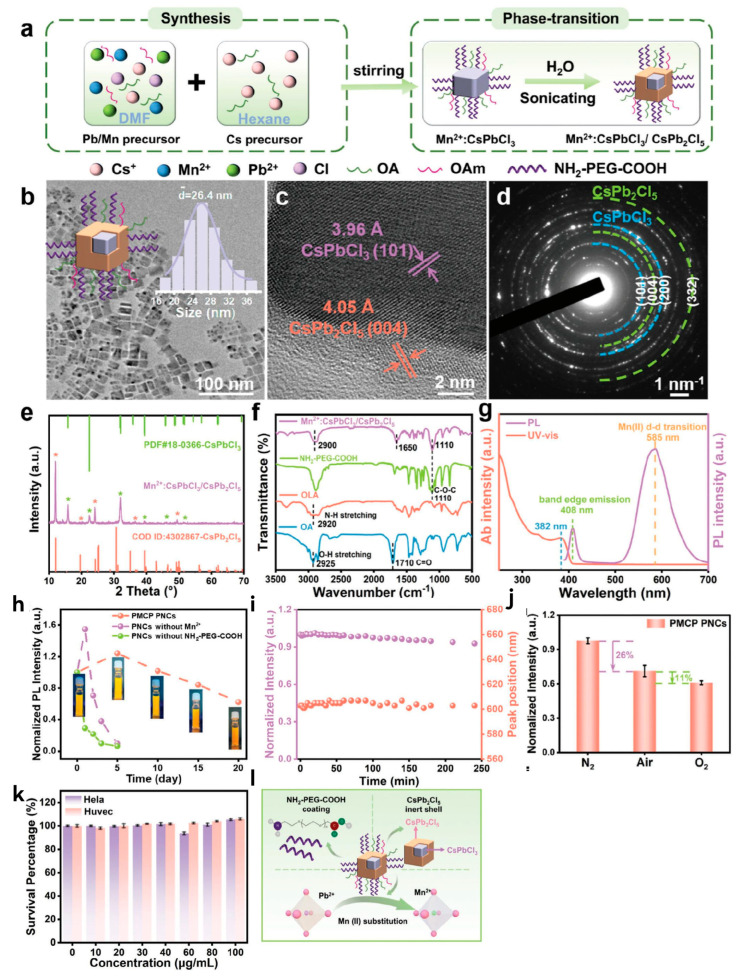
(**a**) Schematic diagram of PMCP PNC formation. (**b**) TEM image and corresponding size distribution of PMCP PNCs. (**c**) HRTEM images of PMCP PNCs. The two crystal lattices show that PNCs have a heterogeneous core/shell structure. (**d**) SAED of PMCP PNCs. The two diffraction rings show the coexistence of CsPbCl_3_/CsPb_2_Cl_5_ heterostructures in PNCs. (**e**) XRD images of PMCP PNCs. (**f**) FTIR spectra of PMCP PNCs, OA, OLA, and NH_2_-PEG-COOH. (**g**) UV-vis spectrum and PL spectrum of PMCP PNCs. (**h**) Storage stability of PMCP PNCs, PNCs without Mn^2+^, and PNCs without NH_2_-PEG-COOH in solutions. (**i**) Water stability of PMCP PNCs under 70 °C heat treatment for 240 min. (**j**) Biological toxicity of PMCP PNCs in Hela and Huvec cells. (**k**) Trinity strategies for improving the stability of PMCP PNCs. (**l**) PL intensity of PMCP PNCs in air, N_2_, and O_2_ environments. Reprinted with permission from ref. [58]. Copyright 2024 WILEY.

It is worth noting that Li et al. recently proposed a novel version of the “trinity strategies” for synthesizing PMCP PNCs that has long-term stability in humid air (Figure 9) [58]. The “trinity strategies” involve three key components: (i) Mn(II) doping to partially substitute Pb(II) and form Mn^2+^:CsPbCl_3_, (ii) NH_2_-PEG-COOH coating, and (iii) water-mediated self-assembly to produce heterostructures. Innovatively, NH_2_-PEG-COOH, as a coating layer, is amphiphilic due to its hydrophilic functional groups (-COOH and -NH_2_), enabling it to provide a protective shell for the internal CsPbCl_3_ NCs [122]. This layer not only shields the crystal structure from excessive water-induced internal erosion but also facilitates the introduction of trace amounts of water molecules. These water molecules can extract CsCl from the outer crystal layers, inducing phase separation to form a CsPbCl_3_/CsPb_2_Cl_5_ core/shell structure [131,132]. Experimental results demonstrate that this material achieves excellent stability, maintaining approximately 60% PLQY even in a pure oxygen environment. Furthermore, it demonstrates low cytotoxicity and good water solubility. This study represents an improvement over the previously proposed “one-pot” synthesis of CsPbCl_3_ NC heterostructures [133] and effectively integrates the advantages of current MHP stabilization strategies. It holds significant value for further exploration in this area.

### 4.4. Shell Passivation Induced by In Situ Phase Change

**Figure 10:**
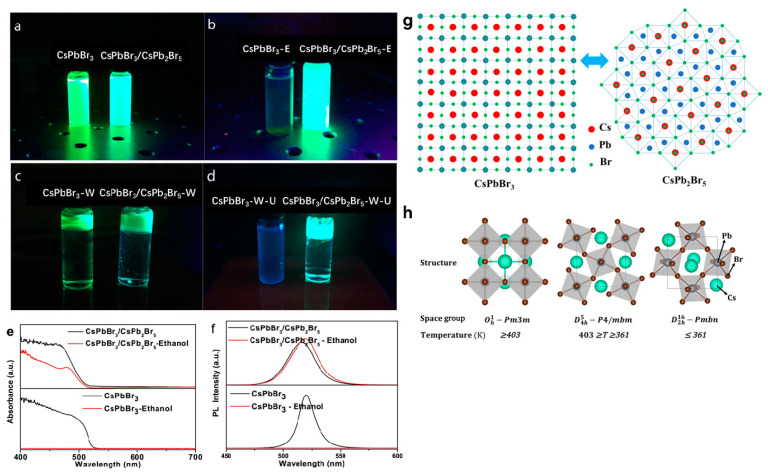
(**a**–**d**) Photographs of CsPbBr_3_ QDs and CsPbBr_3_/CsPb_2_Br_5_ core–shell QDs excited at 365 nm in water and ethanol atmosphere. (**e**,**f**) Absorption spectra and PL spectra of CsPbBr_3_ QDs and CsPbBr_3_/CsPb_2_Br_5_ core–shell QDs, before and after ethanol being added, respectively. (**g**,**h**) The crystal structure of CsPbBr_3_ and CsPb_2_Br_5_. Reprinted with permission from ref. [131]. Copyright 2017 IOP Publishing Ltd.

This method involves in situ external phase separation of MHP QDs induced by water, resulting in a self-assembled core/shell structure. In 2015, Qiao et al. [131] became the first to obtain CsPbBr_3_@CsPb_2_Br_5_ core/shell NCs using a modified non-stoichiometric solution phase method, and by controlling the addition ratio of Cs_2_CO_3_ and PbBr_2_ in the precursor solution. Through quantitative photoluminescence intensity measurements of CsPbBr_3_ and CsPbBr_3_@CsPb_2_Br_5_ in ethanol and water media, they demonstrated a significant improvement in water solubility and stability in aqueous media (Figure 10).

However, Qiao et al. also observed that the phase transition between CsPb_2_Br_5_ and CsPbBr_3_ is reversible. At temperatures exceeding 190 °C, CsPb_2_Br_5_ reverts to CsPbBr_3_ due to the high concentration of Cs^+^ in the system [131]. This reversibility poses challenges for achieving fully encapsulated CsPbBr_3_@CsPb_2_Br_5_ core/shell structures using the commonly employed hot injection method. To address this, Rosa-Pardo et al. [133] controlled the temperature of the precursor solution before hot injection and introduced a rapid quenching step in an ice-water bath. This approach yielded more uniformly coated CsPbBr_3_@CsPb_2_Br_5_ materials. Experimental validation showed that these MHP NCs could maintain high PLQY for up to 18 months in aqueous and DMF media.

Furthermore, Zhang et al. [134] explored the addition of CsPb(OH) layers—a highly stable substance in water—into the CsPbBr_3_/CsPb_2_Br_5_@PbBr(OH) structure by controlling the water content of the reaction system. This approach effectively addressed the stability issues caused by an uneven CsPb_2_Br_5_ coating.

### 4.5. Surface Ligand Exchange and Polymer Shell Encapsulation

Perovskite QDs exhibit excellent optoelectronic properties; however, they are susceptible to environmental factors such as oxygen and moisture, which lead to a degradation of their optical performance. Surface ligands can regulate the surface energy states of PQDs, reducing the recombination of electrons and holes. The electronic and spatial effects of the ligands can both influence the energy level structure of the QDs and improve carrier transport properties. Specifically, certain long-chain ligands can effectively reduce the surface defect density, increasing the PL QYs of the QDs.

**Figure 11:**
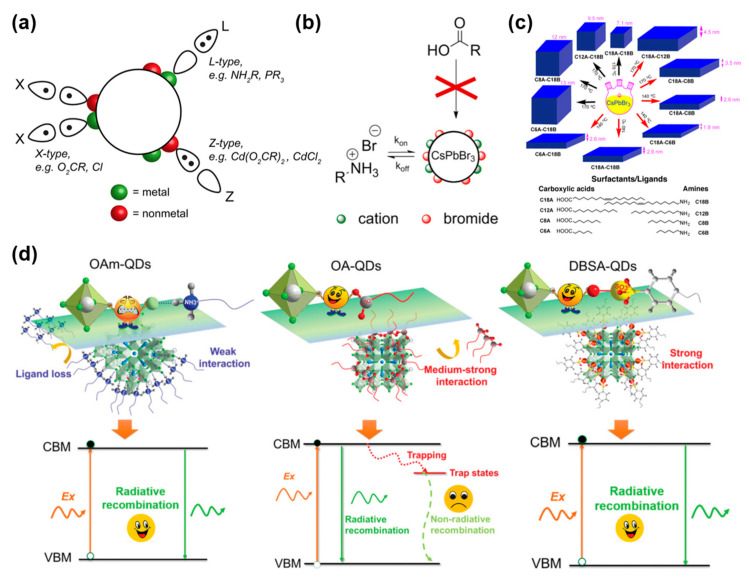
(**a**) Schematic diagrams of X, Z, and L-type ligands. Reprinted with permission from ref. [136]. Copyright2018 American Chemical Society. (**b**) Schematic illustration of the dynamic stability of brominated oleylamine on the surface of PQDs. Reprinted with permission from ref. [138]. Copyright2016 American Chemical Society. (**c**) The effect of different carbon chain lengths on the synthesis of PQDs. (**d**) Schematic diagrams of oleic acid, oleylamine, and sulfonic acid as ligands for PQDs. Reprinted with permission from ref. [140]. Copyright2019 John Wiley and Sons.

Additionally, surface ligands can also modulate the solubility and interface affinity of the QDs. According to Green’s classification of covalent bonds [135], PQD surface ligands are defined as L, X, or Z types based on the number of electrons contributed by the ligand to the metal–ligand bond. L-type ligands are Lewis bases, providing two electrons to the metal–ligand bond. Z-type ligands are Lewis acids, contributing empty orbitals to the metal–ligand bond. X-type ligands provide one electron to form the metal–ligand bond. Figure 11a shows schematic diagrams of X, Z, and L-type ligands.

Organic long-chain molecules, such as oleylamine, are commonly used as ligands in the synthesis of PQDs. Research by Almeida et al. has shown that variations in the ratio of oleic acid to oleylamine affect the system’s acidity and basicity, and under the interaction of both ligands, they jointly determine the size, morphology, and phase control of the perovskite QDs [136]. De Roo et al. [137], through nuclear magnetic resonance (NMR) and NOESY spectra, discovered that oleic acid and oleylamine ligands on the surface of CsPbBr_3_ perovskite QDs exhibit a highly dynamic state (Figure 11b). Figure 11c shows the specific impact of different alkyl ligands on morphology during PQD synthesis. Based on alkyl amines and alkyl carboxylic acids, researchers have progressively developed various organic ligands. Chen et al. [138] replaced oleic acid with conjugated linoleic acid and achieved hydrophobic protection by cross-linking conjugated alkene bonds, thereby enhancing the material’s stability in polar solvent. Yang et al. [139] utilized dodecyl benzenesulfonic acid as a ligand, ensuring that PQDs retained more than 90% of their initial photoluminescence quantum yield (PLQY) even after being stored for five months (Figure 11d). Liu et al. [140] found that by modifying the PQD surface with trioctylphosphine, they achieved a near 100% PLQY, and this optical performance was maintained for over a month.

**Figure 12:**
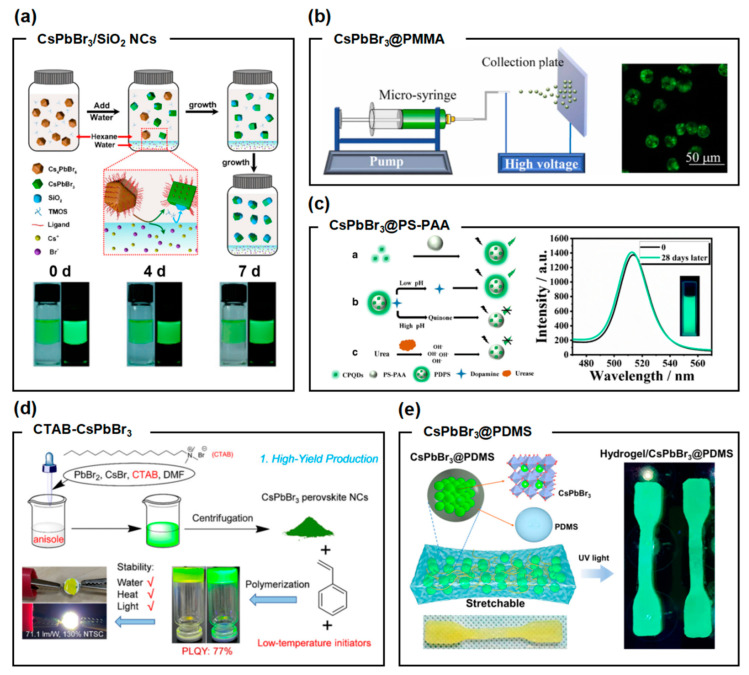
(**a**) Schematic diagram of the formation process of CsPbBr_3_/SiO_2_ NCs and their water resistance stability. Reprinted with permission from ref. [141]. Copyright2023 American Chemical Society. (**b**) Preparation of CsPbBr_3_@PMMA composite fluorescent microspheres via electrospray and their water stability. Reprinted with permission from ref. [142]. Copyright2021 Elsevier BV. (**c**) Water stability of CsPbBr_3_@PS-PAA composites. Reprinted with permission from ref. [143]. Copyright2021 Elsevier BV. (**d**) CTAB-CsPbBr_3_ composites exhibiting enhanced water resistance and thermal stability. Reprinted with permission from ref. [144]. Copyright2021 Springer-Verlag GmbH Germany. (**e**) CsPbBr_3_@PDMS microspheres demonstrating excellent optical performance and stability in water, acid, and alkaline solutions. Reprinted with permission from ref. [55]. Copyright2017 American Chemical Society.

Polymer coating is also an effective method to enhance the water stability of perovskite QDs while maintaining their optical performance (Figure 12). Hu et al. [141] encapsulated monodisperse perovskite nanoparticles with SiO_2_ spheres, significantly improving the stability of the perovskites against water, air, and light exposure. Wang et al. [142] developed a rapid preparation method for CsPbBr_3_@PMMA fluorescent composite microspheres using electrospray, which protects the CsPbBr_3_ QDs from external stimuli and ensures their stability in aqueous environments, with a full width at half maximum (FWHM) of 21 nm. An et al. [143] successfully encapsulated CsPbBr_3_ perovskite QDs in poly(styrene/acrylamide) microspheres using a swelling–shrinking method for simultaneous monitoring of pH, urea, and urease, demonstrating water stability; however, the photoluminescence quantum yield (PLQY) of the PQDs significantly decreased after encapsulation. Cai et al. [144] prepared CTAB-CsPbBr_3_@PS composites, which exhibited enhanced stability against water and heat, but the PLQY was only 77%. Liu et al. [55] synthesized CsPbBr_3_@PDMS microspheres using an emulsion method, showing some stability in water, acid, and alkaline solutions, and further encapsulated them in hydrogels. However, it can be summarized that the surface ligand modifications or coating techniques used in current studies fail to simultaneously ensure good water stability and optical performance (such as PLQY, FWHM, etc.).

## 5. Challenges and Strategies for Decreasing Cytotoxicity of Hydrogel/MHP QD Materials

Among metal halide perovskites, lead-based perovskites are the most common; however, the toxicity of lead limits their widespread application, particularly in biomedical fields. Therefore, replacing lead with non-toxic elements has become a highly prioritized research direction. Considering factors such as atomic radius, relative atomic mass, and electronic configuration, researchers have widely synthesized lead-free perovskite materials using elements such as Sn [145], Bi [146], and Sb [147]. However, the issue of maintaining long-term stability of lead-free perovskite QDs in aqueous environments remains unresolved, and there are few reports on the use of lead-free perovskite QDs in hydrogels. Once the breakthrough of water stability in lead-free perovskites is achieved, more valuable research and applications are expected to emerge.

To decrease the cytotoxicity of hydrogel/MHP QD materials, biocompatible polymers are selected as hydrogel matrices. For example, natural polymers like chitosan and gelatin, and synthetic polymers such as polyethylene glycol (PEG), have good biocompatibility and low cytotoxicity. They can form a stable three-dimensional network structure to encapsulate MHP QDs, reducing the direct contact between MHP QDs and cells and thus decreasing cytotoxicity.

Moreover, the proper surface modification of QDs with ligands or coating materials will help. Ligands with low toxicity and strong binding ability, such as some amino acids and peptides, can be used to replace the original toxic ligands on the surface of QDs. This not only improves the stability of QDs but also reduces their cytotoxicity. In addition, through surface modification with specific ligands, these hydrogel/MHP QDs can be made to home in on particular cell types or tissues with higher precision.

More in-depth biological evaluation studies are lacking in the current state to clarify the interaction mechanism between hydrogel/MHP QD materials and cells, tissues, and organisms at the molecular and cellular levels. This includes studying the uptake, distribution, metabolism, and excretion of materials in vivo to provide a more scientific basis for reducing cytotoxicity. 

## 6. Conclusions and Future Perspectives

In summary, MHP QDs have a high PLQY, narrow FWHM, and easily tunable bandgap. After being embedded in hydrogels, they can still maintain good optical properties, and the resulting hydrogel sensors have high sensitivity to specific substances. Given the exceptional optical properties of MHP QDs and the biocompatibility of hydrogel materials, combined with previous research on fluorescent hydrogels [148,149,150,151,152,153], it is evident that hydrogel/MHP QD composites with enhanced mechanical properties and photonic stability have broad future application prospects. However, the current research on hydrogel/MHP QD materials mainly aims to expand the use of water-stable MHP QDs synthesized in laboratory settings. Their mechanical properties are still far from meeting the requirements of practical applications.

In the future, new strategies will be developed for the hydrogel matrix to protect MHP QDs from environmental factors such as water and oxygen, improving their stability. Through strategies such as the trinity synthesis method, the water stability of perovskite QDs embedded in hydrogels can be further enhanced. Moreover, designing hydrogel/MHP QD materials with superior mechanical properties will require a consideration of multiple issues. For instance, photoinitiated cross-linking is a common method for hydrogel synthesis, but the incorporation of free-radical photoinitiators can generate biologically toxic reactive radicals [154], potentially limiting the biomedical applications of hydrogels. Furthermore, it remains uncertain whether MHP QDs with current core/shell structures can adapt to various hydrogel materials with improved mechanical properties.

We believe that with the deepening of research and the development of new technologies, the continuous improvement of the water stability of MHP QDs, and further breakthroughs in the mechanical properties of hydrogels, the synergistic fusion of the two will become a valuable research target. This integration is designed to meet the high performance and high precision requirements for biomedical applications, such as fluorescent imaging, drug delivery, tissue engineering, phototherapy, and beyond. As technology advances, we anticipate that these hydrogel/MHP QDs will play an even more significant role in personalized medicine. Tailoring their design and function according to individual patient characteristics will open new doors for more effective and safer treatments, potentially revolutionizing the entire field of healthcare.
